# A Precise Framework for Rice Leaf Disease Image–Text Retrieval Using FHTW-Net

**DOI:** 10.34133/plantphenomics.0168

**Published:** 2024-04-25

**Authors:** Hongliang Zhou, Yufan Hu, Shuai Liu, Guoxiong Zhou, Jiaxin Xu, Aibin Chen, Yanfeng Wang, Liujun Li, Yahui Hu

**Affiliations:** ^1^ College of Computer and Information Engineering, Central South University of Forestry and Technology, Changsha 410004, Hunan, China.; ^2^ National University of Defense Technology, Changsha 410015, Hunan, China.; ^3^ Department of Soil and Water Systems, University of Idaho, Moscow, ID 83844, USA.; ^4^ Plant Protection Research Institute, Academy of Agricultural Sciences, Changsha 410125, Hunan, China.

## Abstract

Cross-modal retrieval for rice leaf diseases is crucial for prevention, providing agricultural experts with data-driven decision support to address disease threats and safeguard rice production. To overcome the limitations of current crop leaf disease retrieval frameworks, we focused on four common rice leaf diseases and established the first cross-modal rice leaf disease retrieval dataset (CRLDRD). We introduced cross-modal retrieval to the domain of rice leaf disease retrieval and introduced FHTW-Net, a framework for rice leaf disease image–text retrieval. To address the challenge of matching diverse image categories with complex text descriptions during the retrieval process, we initially employed ViT and BERT to extract fine-grained image and text feature sequences enriched with contextual information. Subsequently, two-way mixed self-attention (TMS) was introduced to enhance both image and text feature sequences, with the aim of uncovering important semantic information in both modalities. Then, we developed false-negative elimination–hard negative mining (FNE-HNM) strategy to facilitate in-depth exploration of semantic connections between different modalities. This strategy aids in selecting challenging negative samples for elimination to constrain the model within the triplet loss function. Finally, we introduced warm-up bat algorithm (WBA) for learning rate optimization, which improves the model’s convergence speed and accuracy. Experimental results demonstrated that FHTW-Net outperforms state-of-the-art models. In image-to-text retrieval, it achieved R@1, R@5, and R@10 accuracies of 83.5%, 92%, and 94%, respectively, while in text-to-image retrieval, it achieved accuracies of 82.5%, 98%, and 98.5%, respectively. FHTW-Net offers advanced technical support and algorithmic guidance for cross-modal retrieval of rice leaf diseases.

## Introduction

Rice, as the core plant in paddy field ecosystems, is an indispensable part of China’s agricultural economy [1]. However, during its growth process, rice is susceptible to various diseases and pests [2], which significantly affect both yield and quality. Diverse leaf diseases such as bacterial leaf blight, rice blast, brown spot, and sheath blight pose significant threats to the normal growth of rice, affecting not only the economic interests of farmers but also the stability of the country’s food production. Currently, with the continuous development of artificial intelligence technology, the application of intelligent methods in controlling leaf diseases is becoming increasingly widespread. However, as technology advances, it has been seen that the limitations of single-modal retrieval are becoming clear. The continuous enrichment of several types of data has led to a growing preference for modern technologies in cross-modal retrieval at the forefront of science and technology. For the prevention and control of rice leaf diseases, cross-modal retrieval technology can rapidly and accurately retrieve comprehensive information about rice leaf diseases. This innovative technology is crucial for prompt and precise disease prevention and control, playing a significant role in promoting the development of the rice industry.

Traditional retrieval methods for crop disease information [3] mainly rely on manual visual inspection. This approach involves seeing the color, texture, insect morphology, and other characteristics of affected crop areas and then comparing them with crop disease image manuals. This method, relying on individual experience and subjective intuition, has significant drawbacks. Although there is a gap in cross-modal retrieval in the agricultural field, in recent years, with the diversification of agricultural data formats [4], scholars have turned to cross-referencing and comprehensive analysis of various modal information. Multi-modal information retrieval technology is currently widely applied in various fields such as healthcare, transportation, and art [5–7], providing valuable references for research in the agricultural direction. In the field of agriculture, the continuous maturity of deep learning technology has enabled intelligent monomodal control of crop diseases [8–12]. However, the data forms of crop diseases show a more diverse modal representation [13]. Models designed solely based on a single modality of images or text are insufficient for more complex disease control. The challenge of adapting to the increasingly diverse problems and challenges in agricultural data, and the limitations of single-modal information (such as images or text) retrieval, have greatly driven the rapid development of cross-modal retrieval in the agricultural field [14,15]. Current existing methods for image–text retrieval can be divided into two major categories: visual semantic embedding methods [16–21] and cross-attention methods [22–27]. In visual semantic embedding methods, these approaches use dual-branch neural networks to map the entire image and text to a shared embedding space. They can enhance inference speed by caching embeddings, achieving excellent efficiency in practical applications. Initially, Frome *et al.* [16] proposed the first model for embedding visual semantics, DeViSE, which used semantic information collected from labeled image data and unlabeled text data to identify visual images. Later, Li *et al.* [17] proposed the selective hard negative mining (SelHN) strategy to address the gradient disappearance issue caused by using only hard negative samples, effectively solving the problem. Additionally, Wang *et al.* [18] developed multilateral semantic relations modeling (MSRM) for image–text retrieval, using hypergraph modeling techniques to capture one-to-many relationships between multiple samples and a given query. Faghri *et al.* [19] proposed the VSE++ method, incorporating online methods into a triplet loss function for challenging negative instance extraction, laying the foundation for further research. Chen *et al.* [20] proposed the VSE∞ framework, which introduces a generalized pooling operator (GPO) to generate best pooling strategies by learning different weights for each ranking dimension. False-negative elimination poses a major challenge, and Li *et al.* [21] introduced an innovative method called false-negative elimination (FNE), including steps for sampling and selecting negative examples, helping to address issues related to FNE. They also designed a momentum-based memory module to keep a large negative buffer and implemented a negative sampling strategy on the buffer, achieving ultramodern performance. On the other hand, cross-attention methods approach the interaction and correlation between text features and image features from the perspective of cross-attention mechanisms. Although these methods perform well in accuracy, their practicality is compromised due to significant computational costs. Initially, Lee *et al.* [22] proposed a model called stacked cross attention (SCAN), selectively aggregating image regions and words to measure image–text similarity. Subsequently, Wei *et al.* [23] incorporated cross-attention and self-attention mechanisms in their work, allowing simultaneous modeling of interactions within and between modalities. In terms of model embedding, Qu *et al.* [24], in their work on dynamic routing mechanisms, proposed a method for adaptively selecting embedding paths based on inputs. They also designed four diverse types of interaction units to enhance interactions within and between modalities. Ge *et al.* [25] introduced the cross-modal semantic enhancement interaction (CMSEI) method, linking intra- and inter-modal semantic relationships between objects and words—a good attempt at perfecting intra-modal interactions. Wei *et al.* [26] designed the multimodality cross attention (MMCA) network to perfect accuracy from both intra- and inter-modal perspectives through a novel cross-attention mechanism. Recently, Zhang *et al.* [27] proposed the negative-aware attention framework (NAAF) method, building on the SCAN method and enhancing similarity calculations by considering the potential adverse effects of mismatched fragments.

During the research, three main challenges were found in current cross-modal retrieval for rice: (a) Complex backgrounds and unclear subject boundaries have long been a focus in the field of image retrieval. These factors often lead to the subject retrieval in images becoming blurry and challenging. Specifically, complex backgrounds may have a lot of interference, making correct subject extraction highly challenging. Moreover, unclear subject boundaries can also complicate feature extraction and may even lead to mistaken retrieval results. (b Enhancing the model’s understanding of diverse text and modeling its relevance has become one of the key issues in current research. In retrieving images with text, a significant challenge lies in the diversity and multitude of text sentences. This results in weaker associations between images and text. In other words, due to the varied forms of text expression, the model needs to have strong semantic understanding capabilities to accurately retrieve images with their corresponding text. (c) Optimizing the learning rate strategy and checking the training process are also critical factors that need careful consideration and design. A well-chosen learning rate strategy can lead the model to efficiently converge toward the global optimum, consequently enhancing the effectiveness and performance of the training process. Additionally, when training retrieval models, we must pay attention to the training convergence speed and the level of accuracy achieved. This relates to the model’s usability and performance in practical applications.

According to the references, we have introduced cross-modal retrieval for the first time in the field of rice. Enhancements have been made to the forefront visual semantic embedding approach, FNE [21], and this framework has been customized for application in the context of rice. Innovatively, we have developed this framework by incorporating advancements in negative sampling strategies, self-attention mechanisms, and learning rate optimization algorithms. Our goal is to construct a highly correct rice leaf disease image–text retrieval framework to address the complexity of rice leaf disease retrieval. This is aimed at providing robust technical support for practical applications in the agricultural sector. Our research aims to ease effective prevention and control of crop leaf diseases and contribute to ensuring food production and promoting the sustainable development of the agricultural industry.

**Fig. 1.**
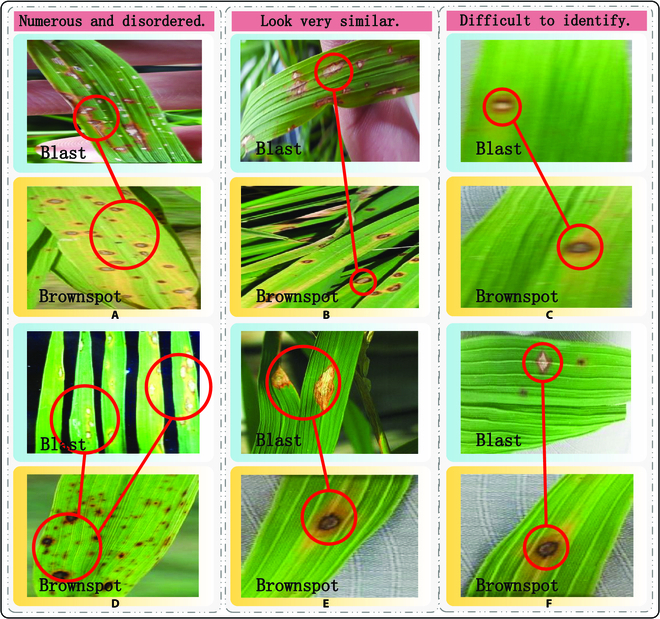
(A to F) Examples of image–text retrieval problems in different disease cases.

For example, diseases such as brown spot and rice blast present as elliptical or irregular lesions of varying sizes (as shown in Fig. 1A), making them challenging to distinguish with the naked eye. They show a high degree of similarity, as illustrated in Fig. 1B. Manual retrieval is prone to errors, reducing accuracy and requiring a significant amount of time and effort. Additionally, manual recognition is susceptible to external environmental factors, such as lighting conditions and fatigue. In image retrieval tasks, when the background is overly complex or the boundaries of the subject are not well defined, it often leads to ambiguity in target retrieval, resulting in retrieval errors. In such cases, due to the presence of background interference, algorithms struggle to accurately decide the position and edge information of the target object, thus affecting the precision and reliability of the retrieval system. Therefore, in such scenarios, enhancing image preprocessing and feature extraction techniques to reduce background interference and improve target retrieval accuracy is crucial for addressing retrieval errors. Consequently, the pursuit of an efficient and correct automated disease retrieval method becomes particularly important. With the rapid development of precision agriculture and smart farming, information related to crop diseases [28] has experienced explosive growth, resulting in exceptionally diverse data. This encompasses a wide range of multimodal data, such as rich images and text, which are often interconnected and complementary. Managing increasingly complex cross-modal data and extracting valuable information from these different modal data, especially those with close semantic relationships, is of paramount importance. Leveraging advanced information technologies like computer vision and image processing to achieve cross-modal retrieval between images and text is vital to meet the ever-growing and diverse demands of individuals seeking crop disease information.

This study addresses significant challenges in cross-modal retrieval of rice leaf diseases. The primary contributions of the research are outlined as follows:

1. To mitigate the severe impact of rice diseases on agricultural production, we focused on four common and highly pathogenic rice leaf diseases, using images and textual descriptions. We created the first cross-modal rice leaf disease retrieval dataset (CRLDRD) dataset and introduced cross-modal retrieval to the field of rice leaf disease retrieval, proposing a framework named FHTW-Net.

2. The complexity of the background or unclear subject boundaries in target images can lead to ambiguity and retrieval errors in image–text retrieval. To further exploit significant semantic information in both images and text, we introduced two-way mixed self-attention (TMS) self-attention to enhance fine-grained feature sequences in images and text, thereby improving model retrieval accuracy.

3. The diversity in the retrieval process, involving diverse text sentences and image morphologies, poses challenges to cross-modal inter-retrieval between images and text. We conducted in-depth research on semantic relationships between different modalities and proposed the FNE-HNM (hard negative mining) strategy, applied to the triplet loss function to enhance the model’s retrieval robustness and generalization capability.

4. In the model training process, insufficient learning rate optimization may result in reduced accuracy and slow convergence in the final retrieval stage. To address this challenge, we introduced warm-up bat algorithm (WBA) to perfect the learning rate, accelerating the model’s training convergence speed and enhancing retrieval accuracy.

5. Experimental results prove that FHTW-Net outperforms existing models. In image retrieval-text task and text retrieval-image task, it achieved accuracies of 83.5%, 92%, and 94% at R@1, R@5, and R@10 for image retrieval, and 82.5%, 98%, and 98.5% for text retrieval. FHTW-Net provides unprecedented cross-modal technical support for timely disease prevention and accurate diagnosis in the field of rice diseases.

## Methods

### CRLDRD dataset

#### Selection and preprocessing of the original dataset

In this study, we used the rice leaf disease image samples (RLDIS) dataset [29] as the source of original images. The dataset forms 5,932 images of rice leaf samples, covering diseases such as bacterial leaf blight, blast, brown spot, and tungro. These images were collected from various rice fields in the western region of Odisha, aiming to capture leaves in different disease states. The areas of lesions on the original leaf images are easily discernible. To keep consistency in the data, all images were resized to 250 × 250 pixels. The dataset underwent a random split, with 80% distributed for training, 10% for validation, and 10% for testing. After annotating text descriptions for all images, the image–text pair dataset, CRLDRD, was used for model training and performance evaluation.

#### Dataset text annotation and construction

**Fig. 2.**
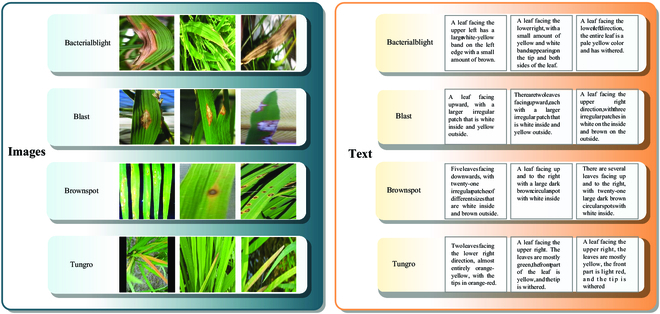
Sample image–text pairs from the CRLDRD dataset.

We enlisted the ability of specialists from the Academy of Agricultural Sciences in Changsha, Hunan Province, China, to provide textual descriptions for the content of the 5,932 images. This process took 80 days. For four diverse types of rice leaf diseases, we conducted detailed annotations of the content of every rice leaf disease image, describing the characteristic features of the diseased leaves, such as spots, colors, disease location, leaf orientation, leaf quantity, and other semantic information that reflects the image features. This eased the establishment of semantic connections between the images and the corresponding text. Such an image–text paired dataset was employed for training the image–text retrieval model, enabling cross-modal retrieval between rice leaf diseases in images and text. Examples of annotations for the four disease categories are illustrated in Fig. 2. In Fig. 2, for images of bacterial leaf blight, we described the distinctive distribution of white spots and the damage on the leaves. For blast disease images, we emphasized the waterlogged condition of the disease spots and their impact on the leaf color. Regarding brown spot disease images, we focused on the brown characteristics of the spots and their distribution on the leaves. For tungro disease images, we described the multiple spots that may appear on diseased leaves and their arrangement.

These detailed textual descriptions not only aid in the model’s understanding of the image content but also provide robust training data for the image–text retrieval model. By setting up such an image–text paired dataset, we have achieved a semantic connection between images and text, offering dedicated support for cross-modal image–text retrieval tasks. The exemplar image–text pairs shown in Fig. 2 are a small part of our annotation work. Each pair of image and text carries rich information, this allows the model to gain a deeper understanding of the features associated with rice leaf diseases, after improving the precision and effectiveness of the image–text retrieval task.

### Model framework

**Fig. 3.**
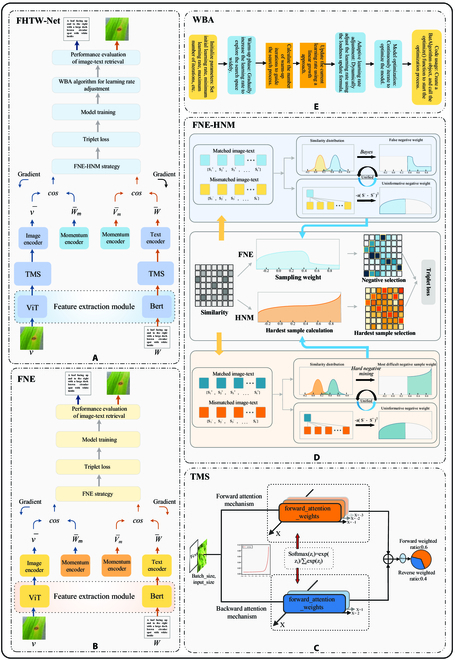
(A to E) Schematic diagram of the proposed FHTW-Net model and its baseline model FNE.

The schematic diagrams of our proposed cross-modal retrieval framework FHTW-Net for rice leaf disease and the baseline model FNE are shown in Fig. 3. In Fig. 3, the model first uses feature extraction module (comprising ViT and BERT) to extract features from image sequences and text sequences, respectively. Next, TMS is applied to enhance the features of both the image sequences and text sequences. The enhanced features are then fed into their respective original feature encoders (image encoder and text encoder) along with their corresponding momentum memory modules (momentum encoder). During model training, the proposed FNE-HNM strategy is employed to comprehensively select and apply challenging negative samples that cut false negatives, and this strategy is applied to constrain the model training with a triplet loss function. Finally, during the training process, we use the proposed WBA to perfect the learning rate, accelerate model convergence, and enhance retrieval performance. Finally, the performance results of FHTW-Net in the image–text retrieval task are presented. Detailed explanations of each module will be provided in later sections. In the “Feature extraction module” section, we will provide a detailed introduction to the feature extraction module. In the “TMS” section, we will describe the details of the proposed TMS. The “FNE-HNM strategy” section will explain the proposed FNE-HNM strategy in detail. In the “WBA” section, we will introduce the proposed WBA algorithm. The contributions of these core modules related to the model will enhance the outstanding retrieval performance and robustness of the cross-modal retrieval framework for rice leaf disease.

#### Feature extraction module

We use the vision transformer (ViT) [30] model for extracting image features and the BERT model for extracting text features. After extracting the image and text features, for the convenience of later loss function optimization and final similarity calculation, we employ average pooling to obtain global features for both the image and text.

**Fig. 4.**
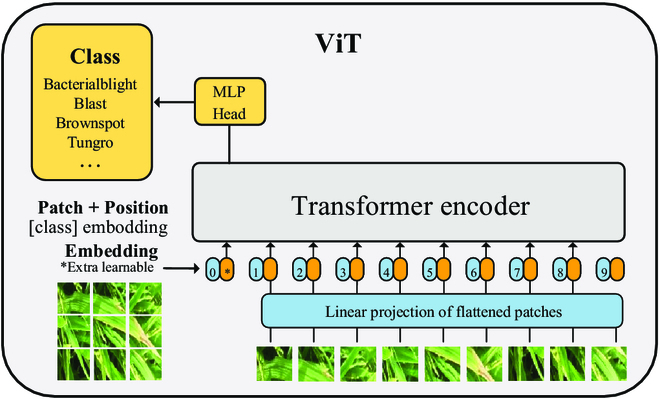
ViT model architecture.

For the selection of the image feature extraction module, we employ the ViT model based on the Transformer architecture [31] as the module for extracting image features. The use of ViT is illustrated in Fig. 3A, and the specific structure of ViT is shown in Fig. 4. In Fig. 4, using multi-head self-attention mechanisms, the ViT model dissects the provided image into smaller blocks, enhancing its capacity to grasp spatial contextual information within the image, then the patch features finally obtained in the image. To enhance the semantic alignment between visual and text modalities, akin to prior investigations [25,32], we project the features of smaller blocks into a common semantic space using fully connected layers. The acquired features of small blocks can be represented as $\bar{v}$ = {*v_i_* |𝑖 = 1, ..., *𝑚*, *v_i_*∈ *R*𝑑}. In Eq. 1, the image representation is demonstrated, with *𝑚* denoting the quantity of small blocks allocated for each image.

$$\bar{v}=\frac{1}{m}\sum_{i=1}^{m}v_{i}\;$$ (1)

**Fig. 5.**
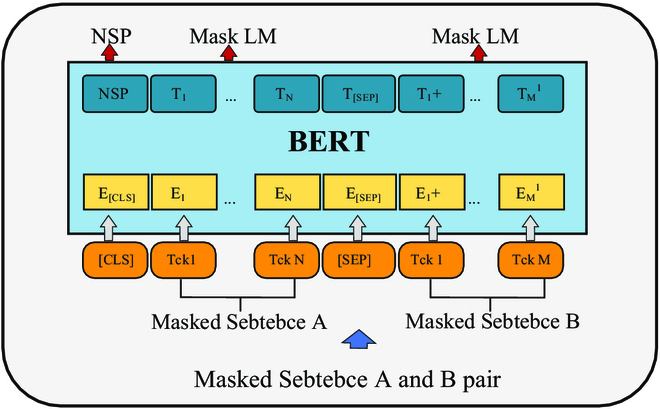
BERT model architecture.

For the selection of the text feature extraction module, to effectively process input text, we follow the latest trends in the field of natural language processing (NLP) and employ a pretrained bidirectional encoder representations from transformers (BERT) [33] model as the module for extracting text features. The use of BERT is illustrated in Fig. 3A, and the specific structure of BERT is shown in Fig. 5. In Fig. 5, BERT is used to extract contextual word representations. Likewise, utilizing fully connected layers, we convert the obtained word features into a unified semantic space, obtaining a feature matrix represented as $\bar{w}$= {*w*𝑗 |𝑗 = 1, ..., 𝑙, *w*𝑗 ∈ *R*𝑑}, where 𝑙 represents the number of words in the sentence and 𝑑 represents the dimensionality of the features. This process aids in capturing the semantic representation of textual information, providing rich text features for our model. The text representation is shown in Eq. 2.

$$\bar{w}=\frac{1}{l}\sum_{j=1}^{l}w_{j}$$ (2)

#### TMS

We propose TMS, aimed at enhancing the representation of features in rice leaf disease images and text sequences. Its specific architecture is depicted in Fig. 3C. The principle of TMS module lies in the use of self-attention mechanisms in different directions and dimensions, allowing the model to address the input data in different directions. Specifically, this module consists of two sets of weights for forward and backward attention, enabling the model to focus on information in different directions of the input data to comprehensively capture features.

In Fig. 3C, it can be seen that TMS first takes a sequence of image features or text features as input. TMS then initializes the weights *forward*_*attention*_*weights* and *backward*_*attention*_*weights* and sets the number of attention heads *Num Heads*. TMS obtains the batch size and hidden size of the input *x* and adjusts the forward and backward attention weights to match the shape of the input *x* through reshaping operations. Next, it calculates the *softmax* weights for forward and backward attention. Forward attention weight calculation involves applying the *softmax* function on different dimensions, specifically on *dim* =  −1, *dim* =  −2, and *dim* =  −3 dimensions. Backward attention weight calculation involves applying the *softmax* function on different dimensions, specifically on *dim* = 1 and *dim* = 2 dimensions, ensuring that the weights form valid probability distributions. Subsequently, using the forward and backward attention weights, it computes the attended features for both directions, obtaining *forward*_*attention*_*weights* and *backward*_*attention*_*weights*. This allows the model to address the input data in different directions. The features addressed in both directions are then linearly combined, with forward weights contributing 60% and backward weights contributing 40%. This weight distribution enables the model to comprehensively consider both forward and backward self-attention, resulting in the final, more comprehensive feature representation *final_features.* Finally, TMS returns the final feature representation *final_features* as the output.

The forward attention weight and backward attention weight calculation formulas for TMS are shown in Eq. 3.

$$\begin{array}{c} \begin{array}{l} \text{forwardweight}\;=\text{soft}\max\left(\text{forward}\_\text{attention}\_\text{weights},\dim=-1\right)\\ \;+\text{soft}\max\left(\text{forward}\_\text{attention}\_\text{weights},\dim=-2\right)\\ \;+\text{soft}\max\left(\text{forward}\_\text{attention}\_\text{weights},\dim=-3\right)\\ \;\text{backwardweight}=\text{soft}\max\left(\text{forward}\_\text{attention}\_\text{weights},\dim=1\right)\\ \;+\text{soft}\max\left(\text{forward}\_\text{attention}\_\text{weights},\dim=2\right) \end{array} \end{array}$$ (3)

Here, *dim* =  −1, *dim* =  −2, and *dim* =  −3 refer to the different dimensions along which the softmax function is applied in the calculation of forward attention weights, while *dim* = 1 and *dim* = 2 refer to the different dimensions in the calculation of backward attention weights. This ensures that the weights form valid probability distributions, allowing the model to address different dimensions of the input data for a more comprehensive feature capture.


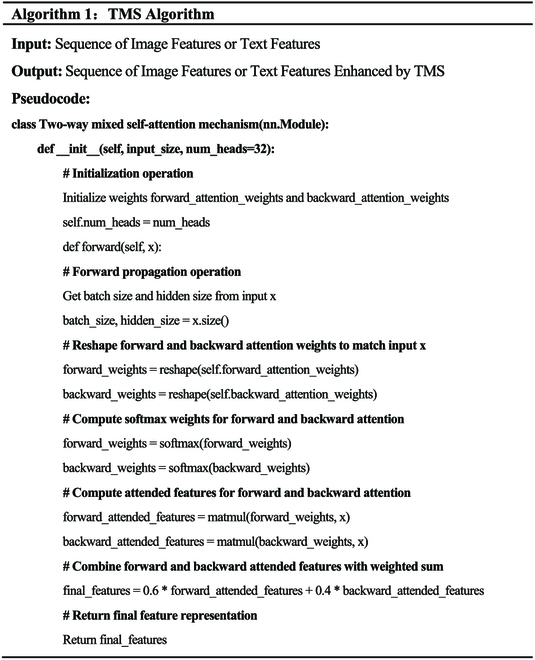


The calculation process for the forward attention features and backward attention features of TMS is described in Eq. 4.

forward_attended_features=matmulforward_weights,x.unsqueeze2.squeeze2backward_attended_features=matmulbackward_weights,x.unsqueeze2.squeeze2 (4)

For the calculation of forward attention features, the forward weights are applied to the input tensor *x* to generate the forward attended features. This involves adding a dimension along the second axis, followed by applying matrix multiplication (*matmul*) with the forward weights to the new dimension. Finally, the dimension is reduced by using *squeeze*(2). The process involves buying the forward attended features through an operation. The computation for backward attention features mirrors that of forward attention features, except it employs the backward weights. These weights are then applied to the input tensor *x*, resulting in the generation of the backward attended features.

The final output calculation process is shown in Eq. 5:

final_features=0.6∗forward_attended_features+0.4∗backward_attended_features (5)

In this equation, the final output calculation combines the features addressed in both forward and backward directions to generate the final feature representation (output). The weight for forward attention is 0.6, while the weight for backward attention is 0.4. This weight distribution allows the model to comprehensively consider both forward and backward self-attention, resulting in a more comprehensive feature representation.

The pseudocode for the feature enhancement of image and text sequences using the TMS module is provided in Algorithm 1.

TMS employs self-attention mechanisms in different directions and dimensions to capture various aspects of input data, enabling the model to more accurately capture the crucial semantic information embedded in both images and text. This allows for effective handling of retrieval errors caused by complex backgrounds or blurred subject boundaries in rice leaf disease image–text retrieval tasks.

#### FNE-HNM strategy

In this section, we will provide a detailed introduction to the FNE-HNM strategy and describe the two integrated strategies within it: the FNE strategy and the HNM strategy. The schematic diagram of the FNE-HNM strategy is shown in Fig. 3D. By combining FNE strategy and HNM strategy through weighted integration, a new strategy called FNE-HNM is proposed to better select negative samples suitable for anchors. The introduction of FNE-HNM aims to integrate the advantages of these two strategies and better balance their influences.

FNE-HNM utilizes the FNE strategy to reduce cases where the model erroneously excludes negative samples that match the anchors. The sampling weight calculation method in the FNE strategy is as follows: first, estimate the probability of each negative sample being falsely negative and, then, apply an exponential activation function to weight the negative samples, where the samples with higher probabilities receive lower weights. FNE-HNM employs the HNM strategy to help the model better differentiate between similar pairs of images and text by training on the most challenging negative examples. The sample selection method using the HNM strategy considers not only the challenge of the samples but also their relative importance.

FNE strategy. In Fig. 3D, Following the extraction of global features from rice leaf disease images and text using the FNE strategy, the alignment between visual and text semantics is managed using triplet loss. At the outset, a metric function is employed to evaluate the distance or resemblance between image pairs $\bar{v}$ and sentences $\bar{w}$ (determining whether they form positive or negative samples). In this context, we use the widely accepted cosine similarity, as defined below:

$$s\left(\bar{v},,,\bar{w}\right)=\frac{{\bar{v}}^{T}\bar{w}}{∥\bar{v}∥∥\bar{w}∥\;}$$ (6)

Subsequently, the triplet loss function is employed to bring positive samples closer and repel negative samples. The formula is as follows:

ℒtri=margin−sv¯,w¯+sv¯,w¯−++margin−sv¯,w¯+sv¯−,w¯+' (7)

where margin is a constraint hyperparameter, being that the distance between a negative sample and the anchor should be greater than margin compared to the positive sample. Failure to adhere to this triplet structure will result in penalties. · + =max(0,·). ${\bar{w}}^{-}$ is text embedding of hard negative samples, and ${\bar{v}}^{-}$ is image embedding of hard negative samples within a small batch.

Many strategies for HNM involve choosing examples that show the highest similarity to the anchor, thus constructing a <anchor, positive sample, negative sample> triplet. As mentioned earlier, these hard negative samples with extremely high similarity may match the anchor due to semantic diversity. In practice, these are referred to as false negatives. However, triplet loss function persists in pushing to increase the separation between them and the anchor. This might introduce ambiguity in the model’s acquisition of semantic representations, resulting in a degradation of performance in image–text matching. An effective remedy for this issue requires the direct exclusion of these falsely identified negative samples during the computation of the triplet loss. However, automating this exclusion proves to be a challenge when explicit labels distinguishing true negatives from false negatives are absent. As a result, we introduce a novel negative sample sampling strategy known as FNE. This strategy aims to tackle the issue by reducing the frequency of false negatives. Specifically, in the case of a negative sample, FNE approach strives to evaluate its posterior probability of being a false negative. This involves the application of Bayesian principles and the incorporation of prior probability distributions for positive and negative samples. As previously mentioned, false negatives pertain to instances labeled as negative in the dataset; however, in terms of meaning, they indeed correspond to the anchor. By trying to gauge the likelihood that a negative sample is a false negative through posterior probability estimation, we are assessing its probability of aligning with the anchor, which is equivalent to the likelihood of being a positive sample. In this pursuit, an examination of the statistical attributes of positive samples is imperative, focusing on matching scores—the degree of similarity between the anchor and the corresponding positive sample. Simultaneously, the computation of the distribution of negative samples is essential, considering that false negatives are categorized as negative samples. Consequently, we derive the similarity values for both positive and negative samples through the following process.

$$\begin{array}{c} S^{+}=\left[s_{1}^{+},s_{2}^{+},s_{3}^{+},\cdots ,s_{i}^{+},\cdots \right]\;\;\;S^{-}=\left[s_{1}^{-},s_{2}^{-},s_{3}^{-},\cdots ,s_{i}^{-},\cdots \right], \end{array}$$ (8)

Here, *S*^+^ and *S*^−^ are defined as sets of similarities for matching and nonmatching pairs, respectively. According to [26], we use normal distributions to model the similarities, as follows:

$$\begin{array}{l} f_{S/c}\left(s\right)=\frac{1}{\sigma^{+}\sqrt{2\pi }}e^{\left[-\frac{{\left(s-\mu^{+}\right)}^{2}}{2{\left(\sigma^{+}\right)}^{2}}\right]}\\ f_{S/\bar{c}}\left(s\right)=\frac{1}{\sigma^{-}\sqrt{2\pi }}e^{\left[-\frac{{\left(s-\mu^{-}\right)}^{2}}{2{\left(\sigma^{-}\right)}^{2}}\right]} \end{array}$$ (9)

In each small batch, the mean (μ+) and standard deviation (σ+) of one distribution, along with the mean (μ−) and standard deviation (σ−) of another distribution, are accumulated and computed. Events *c* and *c¯* represent the stochastic occurrences of matching and nonmatching, respectively. It is important to highlight that we refrain from utilizing pretrained models or preexisting features to form the distributions, given that the model’s representational capacity evolves throughout the training process, static distributions fall short in accurately encapsulating the statistical attributes of similarities essential for effectively tackling false negatives within the present model. When the current model’s capability surpasses it, fixed distributions cannot provide proper hard negative samples and false-negative measurements, limiting further improvement in the current model’s capability. However, since the current model computes similarities, there exists some degree of error. Therefore, to estimate the real distributions of similarity more accurately for matching and nonmatching pairs, we choose to selectively sample the similarities of matching pairs solely from the current small batch when they surpass those of other nonmatching pairs. Subsequently, these sampled similarities are used to calculate the mean and variance, serving as the foundation for constructing the distribution.

After acquiring the initial similarity distributions, expressing the conditional probability of a specific negative sample being a false negative involves representing the posterior probability. *P*(*C* = *c*/*S* = *s*), where *c* signifies the presence of a particular sample aligning semantically with the anchor, while *s* denotes the associated similarity score between them. Nevertheless, given that *C* is a discrete random variable and *S* is a continuous random variable, calculating the conditional probability directly *P*(*C* = *c*/*S* = *s*) is not possible, given that the probability of an event *S* = *s* for a continuous random variable is zero. We attempt to condition not on the event *S* = *s*, but on the event *C* = *c* ∣ *s* ≤ *S* ≤ *s* + *Δs*, where *Δs* represents an infinitely small positive value, proceeding to approach the limit as *Δs* approaches zero. The posterior probability *P*(*C* = *c*/*S* = *s*) is derived using Bayesian rule:

PC=c/S=s≈PC=c/s≤S≤s+Δs=PcPs≤S≤s+Δs/cPs≤S≤s+Δs (10)

In this scenario, *s* signifies the similarity between a particular sample and the anchor. Subsequently, we apply the mean value theorem for integrals.

$$\begin{array}{c} \begin{array}{l} \frac{P\left(c\right)P\left(s\leq S\leq s+\Delta s/c\right)}{P\left(s\leq S\leq s+\Delta s\right)}=\frac{P\left(c\right)\int_{s}^{s+\Delta s}f_{S/c}\left(t\right)\mathrm{dt}}{\int_{s}^{s+\Delta s}f_{S}\left(t\right)\mathrm{dt}}\\ \;\approx \frac{P\left(c\right)f_{S/c}\left(s\right)\Delta s}{f_{S}\left(s\right)\Delta s}\\ \;=\frac{P\left(c\right)f_{S/c}\left(s\right)}{f_{S}\left(s\right)} \end{array} \end{array}$$ (11)

By employing the law of total probability, the denominator *f_S_*(*s*) can be computed in the following manner:

$$f_{S}\left(s\right)=P\left(c\right)f_{S/c}\left(s\right)+P\left(\bar{c}\right)f_{S/\bar{c}}\left(s\right),$$ (12)

where $\bar{c}$ denotes the event of a specific sample not aligning with the anchor. We can express Eq. 13 as:

$$P\left(C=c/S=s\right)=\frac{P\left(c\right)f_{S/c}\left(s\right)}{P\left(c\right)f_{S/c}\left(s\right)+P\left(\bar{c}\right)f_{S/\bar{c}}\left(s\right)}$$ (13)

Derived from the process, the posterior probability *P*(*C* = *c*/*S* = *s*) is influenced by three distributions. In Eq. 14, we have already derived the initial two distributions, which correspond to the similarities of matching and nonmatching pairs. As it is known that for a given sample and anchor there are only two possibilities—matching or nonmatching, this implies that the random variable *C* follows a Bernoulli distribution:

$$C∼B\left(1,p\right)$$ (14)

where *p* is the probability of the event *C*. Given the presence of false negatives in the annotated data, directly computing the conditional distribution parameters *p* for adjustment is not proper. Instead, we adjust the value of *p* and choose the best one derived from the validation set.

After deciding the likelihood of a negative sample being an incorrect negative using Eq. 13, the next step is deciding whether to incorporate this derived false-negative probability into the optimization of the triplet loss. While thresholding is a straightforward approach, it can be nuanced, needing precise tuning of the threshold probability as a hyperparameter. As an alternative, we adopt an intuitive concept: Negative samples with higher probabilities of being false negatives should be less often included in the triplets. We implement this through weighted sampling based on posterior probabilities. To ease a more seamless sampling process, we use the exponential activation function. The entire procedure can be articulated as follows:

$$\mathrm{pi}=\exp\left(\;-\;P\left(C=c\;|\;S=s\left(d_{i}^{-}\;\right)\;\right)\;\right)$$ (15)

Here, *pi* denotes the sampling weight for selecting negative samples, and *di−* is the negative sample. Employing the sampling approach allows for a reduction in the likelihood of meeting false negatives. Nevertheless, to focus the optimization effort on hard negative samples, we implement a reduction strategy for easy negative samples. These samples prove an incorrect negative likelihood approaching zero and should be excluded from the sampling process using the FNE strategy. This is supported by the observation that these negative samples are unrelated to the anchor and unmistakably do not correspond to it, as highlighted in [26,34,35]. Including these easily distinguishable negative samples in the triplets does not contribute valuable information for model optimization. Therefore, for negative samples with incorrect negative likelihoods approaching zero, we reduce their sampling weights and reset them according to the following procedure:

$$\mathrm{pi}=\exp\left(-a\left(s{\left(s\left(d_{i}^{-}\right)-s\left(d^{+}\right)\right)}^{2}\right)\;\right)$$ (16)

Here, *a* is a hyperparameter dictating the sampling density. The negative sampling in FNE strategy combines these two methods. Equation 17 can be articulated as follows:

$$\begin{array}{c} p_{i}=\left\{\begin{array}{c} \;exp\left.\left(-P(C=c|S=s\left(d_{i}^{-}\right)\right)\right),\;\;\;others\\ exp\left(-a{\left(s\left(d_{i}^{-}\right)-s\left(d^{+}\right)\right)}^{2}\right),\;\;\;P\left(C=c|S=s(d_{i}^{-})\right)\leq \lambda^{2} \end{array}\right. \end{array}$$ (17)

Here, *λ* is configured to be 0.01. This approach ensures that false negatives and easily identifiable negative samples incur lesser penalties in terms of sampling weights. This adjustment enables the model to focus on learning semantic representations primarily from genuine and more challenging negative samples.

Through the FNE strategy, we can eliminate negative samples of rice leaf diseases with false negatives, addressing the inter-class similarity issue among different diseases in rice cross-modal image–text retrieval.

HNM strategy. In Fig. 3D, HNM strategy is applied to the triplet loss to select the most challenging negative examples, accelerating training and improving model performance. The workflow of HNM strategy in Fig. 3D is as follows.

Step 1: Compute similarity scores. Here, the similarity scores between the image embedding matrix (*im*) and the text embedding matrix (*s*) will be calculated. This is achieved through a matrix dot product operation, denoted by @*.* The formula is as shown in Eq. 18.

scores=im@s.T (18)

Step 2: Calculate diagonal scores. Next, the model computes the diagonal elements of the similarity score matrix, which are respectively stored in *d*1 and *d*2 .These diagonal elements are the similarity scores of each image or text with itself. We can rewrite Eq. 19 as:

$$\begin{array}{c} \begin{array}{c} diagonal|=scores.diag().view(im.size(0),1)\\ d1|=diagonal.expand\_as(scores)\\ d2|=diagonal.t().expand\_as(scores) \end{array} \end{array}$$ (19)

Step 3: Compute retrieval loss for text and image. In this step, the model calculates the retrieval losses for text *cost*_*s* and image *cost im*. These losses are related to the similarity scores, diagonal scores, and a hyperparameter margin. The loss values are compared with zero using the clamp function to ensure that they are not less than zero. We can rewrite Eq. 20 as:

$$\begin{array}{c} \begin{array}{c} \cos t\_s|=(margin+scores-d1).clamp(min=0)\\ \cos t\;im|=(margin+score-d2).clamp(min=0) \end{array} \end{array}$$ (20)

Step 4: Clear values on the diagonal. In this step, a mask matrix is created to clear the values on the diagonal of the loss matrix. This is done because the loss values on the diagonal have no meaning for the similarity between each sample and itself. We can rewrite Eq. 21 as:

$$\begin{array}{c} \begin{array}{c} mask\;=torch.eye(scores.size(0))>0.5\\ \cos t\_s\;=\cos t\_s.masked\_\;fill\_(mask,0)\\ \cos t\_im\;=\cos t\_im.masked\_\;fill\_(mask,0) \end{array} \end{array}$$ (21)

Step 5: Implement HNM strategy. Next, the HNM strategy is implemented. For text retrieval, the model selects the hardest negative samples for each query (each row) by finding the maximum of (*margin* + *scores* − *d*1). For image retrieval, the model selects the hardest negative samples for each image (each column) by finding the maximum of (*margin* + *scores* − *d*2). We can rewrite Eq. 22 as:

$$\begin{array}{c} \begin{array}{c} hard\_neg\_s|=(margin+scores-d1).max(1)\left[0\right]\\ hard\_neg\_im|=(margin+scores-d2).max(0)\left[0\right] \end{array} \end{array}$$ (22)

Step 6: Calculate the final triplet loss. Finally, the ultimate triplet loss *triplet*_*loss* is computed, which is a combination of text retrieval loss, image retrieval loss, and HNM strategy loss. By summing these losses and subtracting the sums of scores from Hard Negative samples, the final loss value is obtained. We can rewrite Eq. 23 as:

$$\begin{array}{c} \begin{array}{c} \text{triplet}\_\text{loss}=\text{cost}\_s.\mathrm{sum}\left(\right)+\text{cost}\_\mathrm{im}.\mathrm{sum}\left(\right)\\ \;-\text{hard}\_\mathrm{neg}\_s.\mathrm{sum}\left(\right)-\text{hard}\_\mathrm{neg}\_\mathrm{im}.\mathrm{sum}\left(\right) \end{array} \end{array}$$ (23)

Applying the HNM strategy to the triplet loss accelerates model training and improves performance. This strategy helps select the most challenging negative samples of rice leaf diseases. Such a negative sample selection strategy aids FHTW-Net in better addressing the inter-class similarity issue among different diseases in rice cross-modal image–text retrieval, enhancing the model’s retrieval performance.

Application of FNE-HNM Strategy. FNE-HNM is applied to the triplet loss during the training phase of the model, while it is no longer executed during the model validation and inference phases. During the model training phase, in addition to the existing methods [17,19,22,25,35], we incorporate the proposed FNE-HNM strategy into the triplet loss to impose constraints on the model. Contrary to earlier studies, the unfavorable instances appointed for exclusion are not anymore limited exclusively to challenging adverse instances. Instead, they are sampled utilizing FNE-HNM strategy, which can eliminate false negatives while remaining challenging. The final triplet loss function with the application of FNE-HNM strategy is as follows.

$$\begin{array}{c} ℒHNM=[margin-s(\bar{v},{\bar{w}}_{fixHNM}){]}_{+}\\ +[margin-s(\bar{v},\bar{w})+s({\bar{v}}_{fixHNM},\bar{w}){]}_{+}, \end{array}$$ (24)

$$\begin{array}{c} ℒFNE=[margin-s(\bar{v},\bar{w})+s(\bar{v},{\bar{w}}_{fixFNE}){]}_{+}\\ +[margin-s(\bar{v},\bar{w})+s({\bar{v}}_{fixFNE},\bar{w}){]}_{+}, \end{array}$$ (25)

$$\begin{array}{c} ℒ\text{total}=\text{alpha}*ℒ\mathrm{HNM}+\left(1-\text{alpha}\right)*ℒ\mathrm{FNE} \end{array}$$ (26)

In the triplet loss function, ℒHNM is the *HNM* loss. The purpose of this loss function is to penalize the model for misclassifying hard-to-classify negative samples. Specifically, *margin* is a threshold used to measure whether the distance (score) between positive and negative samples is large enough. $s\left(\bar{v},,,\bar{w}\right)$ is the score output by the model, where $\bar{v}$ is the model’s prediction for the sample, and $\bar{w}$ is the true label of the sample. Here, ${\bar{w}}_{\text{fixHNM}}$ and $\;{\bar{v}}_{\text{fixHNM}}$ respectively represent the negative embeddings of text and image selected by our *HNM* strategy. *alpha* is a weight parameter used to control the weight of ℒHNM loss in the total loss. ℒFNE is the FNE loss. The purpose of this loss function is to penalize the model for misclassifying false-negative samples. Like ℒHNM, the difference lies in the use of *_fixFNE_* samples. Here, ${\bar{w}}_{\mathrm{fixFNE}}$ and ${\bar{v}}_{\mathrm{fixFNE}}$ respectively represent the negative embeddings of text and image chosen through our FNE strategy. *alpha* is also used here to control the weight of ℒFNE loss in the total loss. Finally, ℒtotal is the overall loss function composed of ℒFNE and ℒHNM, where *alpha* controls the weight allocation between the two. During training, we adjust the model’s weights by minimizing ℒtotal to better classify positive and negative samples, especially for hard-to-classify negative samples and false negatives. This helps improve the model’s performance and robustness.

During the model validation and inference phases, the FNE-HNM negative sample sampling strategy is no longer executed. Instead, the focus is on using similarity rankings to obtain the most relevant samples, thereby improving the efficiency and accuracy of inference. Our framework follows a conventional approach, wherein the resemblance between the query anchor and all samples in the test set is computed. Followed by ranking them according to their similarity scores. The top-ranked samples, such as R@1, R@5, and R@10, are considered retrieval results. These results are the samples that the model considers most like the query anchor, which are used for our rice cross-modal image–text retrieval task.

#### WBA

We propose WBA to perfect the learning rate of the FHTW-Net retrieval model. This aims to accelerate the convergence speed of the model and improve the retrieval accuracy. The principal diagram of the WBA is shown in Fig. 3E. The learning rate, as a crucial hyperparameter, controls the update step size of model parameters. However, traditional learning rate adjustment strategies may not be flexible enough in some cases, especially when the model parameter space is unstable or highly nonconvex. Therefore, we propose this adaptive learning rate optimization method. The WBA dynamically adjusts the learning rate to adapt to various stages of model training. The core idea of the algorithm is to adaptively adjust the learning rate based on the progress of training iterations. In the first stages of training, the algorithm performs a warm-up process by setting the learning rate to a larger first value to quickly approach the global optimum. As training progresses, the learning rate gradually decreases to stabilize the convergence of model parameters. This adaptiveness helps overcome potential training difficulties that may be met with traditional fixed learning rate strategies.


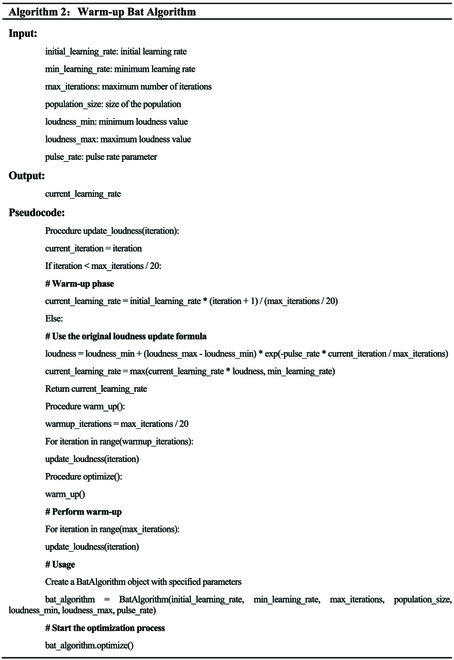


In Fig. 3E, WBA first initializes the parameters, then enters the warm-up phase for learning rate updates. At the beginning of the algorithm, it enters a stage called the warm-up period. In this stage, the goal of the algorithm is to gradually guide the search process, starting from the first state, and gradually increasing the learning rate to explore the search space more extensively. The duration of this warm-up period accounts for 1/20 of the total number of iterations. The algorithm first calculates the number of iterations for the warm-up period, as shown in Eq. 27.

$$\begin{array}{c} Warmup\_iterations=\frac{max\_iterations}{20} \end{array}$$ (27)

For each iteration within the range from 0 to *warmup*_*iterations*, the algorithm updates the current learning rate in a linearly increasing manner, as shown in Eq. 28 below.

$$\begin{array}{c} \begin{array}{c} \text{Current}\_\text{learning}\_\text{rate}=\text{initial}\_\text{learning}\_\text{rate}\\ \;*\frac{\text{iteration}+1}{\text{warmup}\_\text{iterations}} \end{array} \end{array}$$ (28)

Here, *Current*_*learning*_*rate* represents the current learning rate, *initial*_*learning*_*rate* is the initial learning rate, *warmup*_*iterations* represents the current iteration number, and *warmup*_*iterations* is the number of iterations for the warm-up period.

Equation 28 indicates that in the first 1/20 of iterations, the learning rate will rapidly approach a larger value in a linearly increasing manner to accelerate the convergence speed of the model. The goal of this process is to explore with a smaller learning rate in the first stages of the search, gradually guiding the search into more promising regions.

Next, it enters the stage of learning rate updates using the Bat Algorithm [36]. Once the warm-up period ends, the algorithm proceeds to the loudness adjustment stage. In this stage, the algorithm uses a loudness value to control the variation of the learning rate for a finer-grained search. The process of loudness adjustment is as follows. The algorithm calculates the change in loudness and is computed according to the loudness update Eq. 29 below.

$$\begin{array}{c} Loudness=loudness\_min+\left(loudness\_max-loudness\_min\right)\\ {}*exp\left(-pulse\_rate*current\_iteration/max\_iterations\right) \end{array}$$ (29)

Here, *Loudness* represents the loudness, *loudness*_*min* and *loudness*_*max* are the minimum and maximum values of loudness, pulse_rate is the pulse rate parameter, *current*_*iteration* represents the current iteration number, and max_*iterations* are the maximum number of iterations.

Next, the algorithm uses the loudness value to update the learning rate, calculated as shown in Eq. 30 below.

$$\begin{array}{c} \text{Current}\_\text{learning}\_\text{rate}=\\ \max\left(\text{current}\_\text{learning}\_\text{rate}*\text{loudness},\min\_\text{learning\_rate}\right) \end{array}$$ (30)

Here, *Current*_*learning*_*rate* represents the current learning rate and min_*learningrate* is the minimum learning rate.

Equation 30 ensures that the learning rate will not go below the minimum value and adjusts dynamically based on the changing loudness. The adjustment of the loudness value leads to dynamic changes in the learning rate during the search process, adapting to the nature of the problem and the progress of the search.

The pseudocode for the Bat Algorithm learning rate optimization is shown in Algorithm 2.

Finally, the optimization of the WBA takes place. In this optimization phase, the model enters the main search and optimization stage. In this critical stage, the algorithm relies on dynamic learning rates to guide the search for the best solution to the problem. In each iteration, the algorithm performs one of the following operations based on the current learning rate: Generate a new solution: The algorithm may generate an entirely new solution based on the current learning rate to expand the search space and find better candidate solutions. Optimize the current solution: Another possibility is that the algorithm perfects the current solution based on the current learning rate to further improve the existing solution. In the optimization phase, WBA continuously tries different solutions until it reaches the predetermined maximum number of iterations (*max*_*iterations*) and stops.

The proposed WBA effectively addresses the potential shortcomings of learning rate optimization, which may lead to low precision and slow convergence speed of the model in the final retrieval stage. WBA accelerates the convergence of the FHTW-Net model training and improves the accuracy and robustness of rice leaf disease image–text retrieval.

## Experiments

### Experimental environment setup

All experiments in this study were conducted in the same hardware and software environment to avoid different experimental conditions affecting the results of FHTW-Net. In this experiment, the NVIDIA GeForce RTX A5000 GPU (24G) was primarily used for training. While the versions of PyTorch, Python, and CUDA do not affect the experimental results, they must be compatible with specific software and hardware. FHTW-Net was built using PyTorch 1.10, Python 3.8, and CUDA 11.3.

The size of input images was uniformly adjusted to 256 × 256 pixels. A total of 5,932 pairs of image–text data were input. In this study, we used fivefold cross-validation for training to rigorously evaluate the model’s performance while avoiding overfitting. Therefore, all input data were randomly divided into training, validation, and test sets in an 8:1:1 ratio. The training set was used to train the model, the validation set was used to evaluate the performance and parameters of the trained model and to select the best model during the training process, and the test set was used to evaluate the final performance of the model.

During training, we set the batch size to 32, and the size of the momentum memory module was set to 8,192. A total of 110 epochs of training were conducted. To perfect the model’s learning rate, we applied the proposed WBA with an initial learning rate of 0.00004. This algorithm adapts the learning rate during training and undergoes reasonable decay to ensure that the retrieval performance of the model converges to the global optimum.

We used the base versions of pretrained BERT and ViT models, specifically bert-base-uncased and vit-base-patch16. These models offer strong foundational feature extraction capabilities, proving helpful for our task. Concerning the loss function, we employed the FNE-HNM strategy on the triplet loss function and pragmatically set up the margin value to 0.2. This helps adjust the training aim of the model to better suit our needs. Moreover, we incorporated the hyperparameter 𝛼 from Eq. 16 to fine-tune the sampling density, setting it to 0.5. The choice of this parameter involved meticulous experimentation and tuning to achieve best performance.

### Evaluation metrics

For the evaluation of FHTW-Net on the CRLDRD dataset, following the approach in [37], we utilized *R*@ *K* (*R*@*K*) as the evaluation metric for retrieval results, where *K* = 1, 5, 10. Recall@K measures the percentage of queries where the true answer is found within the top *K* ranked list. A higher *R*@*K* value shows better performance.

*R*@*K* evaluates the percentage of correct answers that appear in the top *K* positions in the ranked list for each query. The computation of this metric is shown in Eq. 31:

R@K=TPTP+FN (31)

Here, (True Positives) is the number of correct hits within the top *K* positions, and (False Negatives) is the number of true answers that were not found within the *K* top position.

In the context of rice leaf disease cross-modal image–text retrieval task, we used different values of *K* (*K* = 1, 5, 10) to assess the model’s performance. This approach is crucial because different *K* values reflect various aspects of the retrieval results related to rice leaf diseases. The choice of *K* is pivotal in this task, as different retrieval scenarios demand different outcomes. When *K* = 1, the focus is on quickly finding the most relevant result, which is crucial for urgent situations in rice leaf disease detection. On the other hand, when *K* = 10, a more comprehensive set of information is obtained to support in-depth research and analysis, such as long-term monitoring of rice leaf diseases. In summary, different values of *K* cater to the requirements of diverse application scenarios, ensuring that the model can deliver retrieval results that meet the needs of agricultural applications in various situations.

### Module effectiveness experiment

This section provides a detailed analysis of the evaluation metrics and parameters for TMS, FNE-HNM, and WBA. The effectiveness of each innovative module was confirmed under the same experimental settings. Below is the presentation of experimental results.

#### Effectiveness of TMS

We propose TMS to enhance the feature sequences extracted by ViT and BERT for images and texts, respectively. The effectiveness experiment results are listed in Table 1. We compared TMS with other self-attention mechanisms, namely, shared self-attention [38], adaptive self-attention [39], sparse self-attention [40], and global-local self-attention [41]. Experimental results show that TSM can improve the accuracy of rice leaf disease image–text retrieval. In comparison with Shared Self-Attention and Sparse Self-Attention, Our TMS demonstrates the most significant improvement. This is because our TMS utilizes self-attention mechanisms in different directions and dimensions, allowing the model to focus on semantic information from various directions and dimensions of the input rice leaf disease image–text pairs. This achieves the best feature enhancement results. The ability of TMS effectively addresses complex background interference in rice leaf disease image–text retrieval, thereby improving the accuracy of the image–text retrieval.

**Table 1.** Effectiveness verification of TMS module on the test set

Methods	Images to text			Text to images		
R@1	R@5	R@10	R@1	R@5	R@10
FNE [21]	80.3	89.8	92.6	79.7	96.2	96.7
FNE + Shared self-attention [38]	80.8	90.0	92.8	79.8	96.3	96.8
FNE + Adaptive self-attention [39]	80.7	90.2	92.9	80.0	96.4	97.1
FNE + Sparse self-attention [40]	80.6	90.1	92.8	79.9	96.3	96.9
FNE + Global-local self-attention [41]	80.9	90.2	92.9	80.0	96.5	97.0
Ours (FNE + TMS)	81.6	90.7	93.2	80.3	96.8	97.3

#### Effectiveness of FNE-HNM

**Fig. 6.**
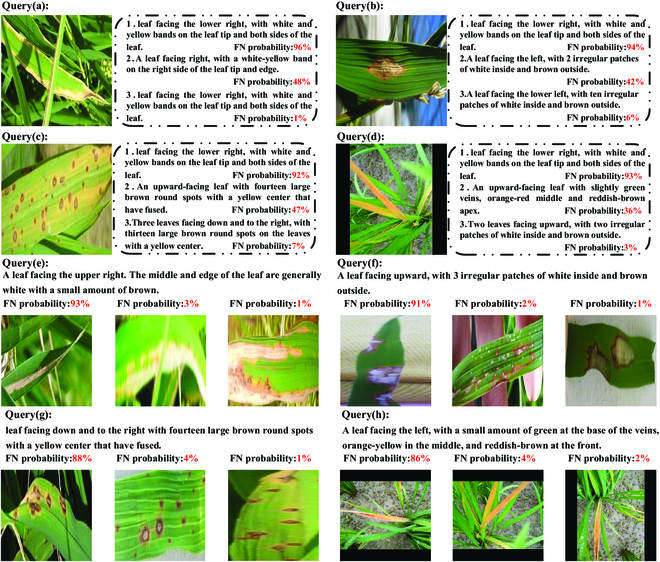
Examples of checking false-negative probability of negative samples when implementing FNE-HNM strategy. FN: the probability pertains to the false-negative probability.

We propose FNE-HNM to select negative samples for the triplet loss function, and the effectiveness experiment results are shown in Fig. 6. In Fig. 6, we evaluate the estimated false-negative probability of negative samples selected by the FNE-HNM strategy. The purpose of checking the false-negative probability of negative samples is to ensure that our FNE-HNM strategy, after selecting challenging negative samples through the HNM strategy, can eliminate false negatives of negative samples through the FNE strategy. The implementation of the FNE-HNM strategy allows the FHTW-Net model to select comprehensive negative samples that are both challenging and free of false negatives for model training, thereby accelerating model training speed and improving retrieval accuracy. We present several examples of false-negative checks in Fig. 6. The examples at the top correspond to image anchors, while the examples at the bottom correspond to text anchors. Within the dataset, every instance comes with an associated false-negative probability and is classified as a negative sample. However, upon observation, certain negative samples closely correspond in semantics to the respective anchor and should ideally be paired with it. In prior research, owing to their substantial similarity to the anchors, these negative samples were identified as challenging negative instances. Consequently, they were specifically targeted for separation using the HNM strategy, leading to conflicting optimization objectives. By adopting FNE-HNM, as illustrated in the diagram, we can evaluate the likelihood of negative samples transitioning into false negatives, referred to as the false-negative probability. Subsequently, we deploy a weighted sampling approach to decrease instances in the triplets. Moreover, it is clear that instances with low incorrect negative probabilities indeed display different meanings from the anchor. In summary, FNE-HNM effectively estimates probability of negative samples becoming false negatives while addressing the challenge posed by negative samples. The FNE-HNM strategy can select comprehensive and challenging negative samples, effectively mitigating the inter-class similarity among distinct categories of rice leaf diseases. This breakthrough can enhance the performance of rice leaf disease image–text retrieval.

#### Effectiveness of WBA

**Fig. 7.**
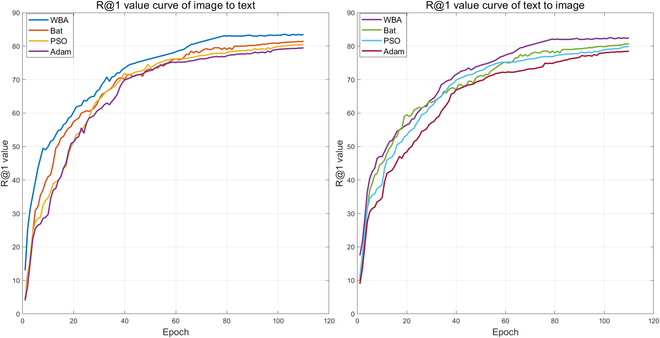
Curve of R@1 value changes during training.

We propose WBA to perfect the learning rate of the rice leaf disease image–text retrieval model FHTW-Net. We evaluate the effectiveness of WBA for learning rate optimization, as shown in Fig. 7. For learning rate optimization, aiming to achieve the goal of flexible, intelligent, and rapid adjustment of the learning rate, we drew inspiration from the Bat Algorithm [36], which simulates bat search, and proposed the improved WBA. We compared our WBA algorithm with learning rate optimization using the bat algorithm, particle swarm optimization (PSO) [42], and the Adam algorithm [43] from FNE. In Fig. 7, we pay special attention to how the retrieval accuracy (R@1) of model training changes with training epochs to evaluate the effectiveness of each optimization algorithm. The experimental results confirm why we proposed the WBA algorithm and its advantages.

From Fig. 7, it can be clearly seen that the performance of WBA far surpasses the other algorithms. In the first training epochs, after a few rounds, it quickly converges and achieves a high accuracy, while the performance of other algorithms is comparatively poorer at the same time. PSO shows a slightly insufficient performance in dynamic adaptation of the learning rate, leading to a lower trend in R@1 values. Adam algorithm also shows a lower trend due to insufficient task adaptability. This shows that the WBA performs better in optimizing the model’s learning rate, allowing the model to converge to a higher performance level more quickly, resulting in retrieval performance superior to other learning rate optimization algorithms.

This practical effect is one of the key reasons for proposing the WBA as our optimization algorithm. In addition to this, we also saw that in the long-term training process, the variation trend of the R@1 value during training with the original Bat Algorithm is highly fluctuating. On the other hand, our improved WBA keeps a higher level of stability. The variation of R@1 during the model training process is smoother compared to other optimization algorithms. This reduces the risk of overfitting, and compared to the Adam algorithm used by the FNE model, the improved convergence stability proves a more powerful performance. This further reinforces our choice.

In summary, based on comprehensive experimental comparisons and the results of R@1 accuracy change curves, it has been confirmed that the proposed WBA algorithm is applicable to the specific rice leaf disease image–text retrieval task. WBA shows better first state and dynamic learning rate adaptability in learning rate optimization, significantly enhancing the retrieval performance of FHTW-Net and the convergence speed of model training.

### Ablation study

Extensive ablation studies were performed on the CRLDRD dataset to assess the impact of each novel element in the proposed approach. The outcomes, obtained from a single model, are displayed in Table 2. Based on these findings, we make the following observations.

**Table 2.** Ablation experiment results of the effectiveness of innovative modules

Methods	Images to text			Text to images		
R@1	R@5	R@10	R@1	R@5	R@10
FNE [21]	80.3	89.8	92.6	79.7	96.2	96.7
FNE + TMS	81.6	90.7	93.2	80.3	96.8	97.3
FNE + HNM	81.3	90.4	93.0	80.1	96.6	97.1
FNE + WBA	81.2	90.2	93.1	80.0	96.5	97.0
FNE + TMS + HNM	82.7	91.2	93.5	81.3	97.4	97.9
FNE + TMS + WBA	82.5	91.0	93.3	81.1	97.2	97.6
FNE + HNM + WBA	82.3	90.7	93.2	80.9	97.0	97.4
Ours (FHTW-Net)	83.5	92.0	94.0	82.5	98.0	98.5

When using only the FNE baseline model and removing TMS, FNE-HNM, and WBA, there is a sharp drop in performance. This verifies the effectiveness of modules such as TMS, FNE-HNM, and WBA in improving model performance. When only FNE-HNM is removed, performance drops slightly further. This is because FNE-HNM expands the pool of negative samples while increasing the challenge of collecting negative samples. The FNE-HNM strategy, an improvement on the baseline FNE strategy, enables more comprehensive sampling and use of challenging yet false-negative-free negative samples for training, providing a more comprehensive and meaningful set of negative samples. This enhancement leads to improved retrieval accuracy and robustness of the model. When only TMS is removed, performance drops slightly further. This shows that TMS enhances both image and text features, playing a role in feature enhancement. This allows the model to achieve better semantic alignment between images and text, thereby enhancing image–text retrieval performance. When only the WBA is removed, the convergence speed of the model decreases, and performance slightly drops. This shows that WBA, through intelligent adaptive search of the learning rate, allows the model to iterate to the best solution more quickly. The more advanced learning rate decay strategy enables the model to achieve better performance. The quantitative analysis results fully reflect the improvement and enhancement of several innovative modules on our model’s performance and training speed.

**Fig. 8.**
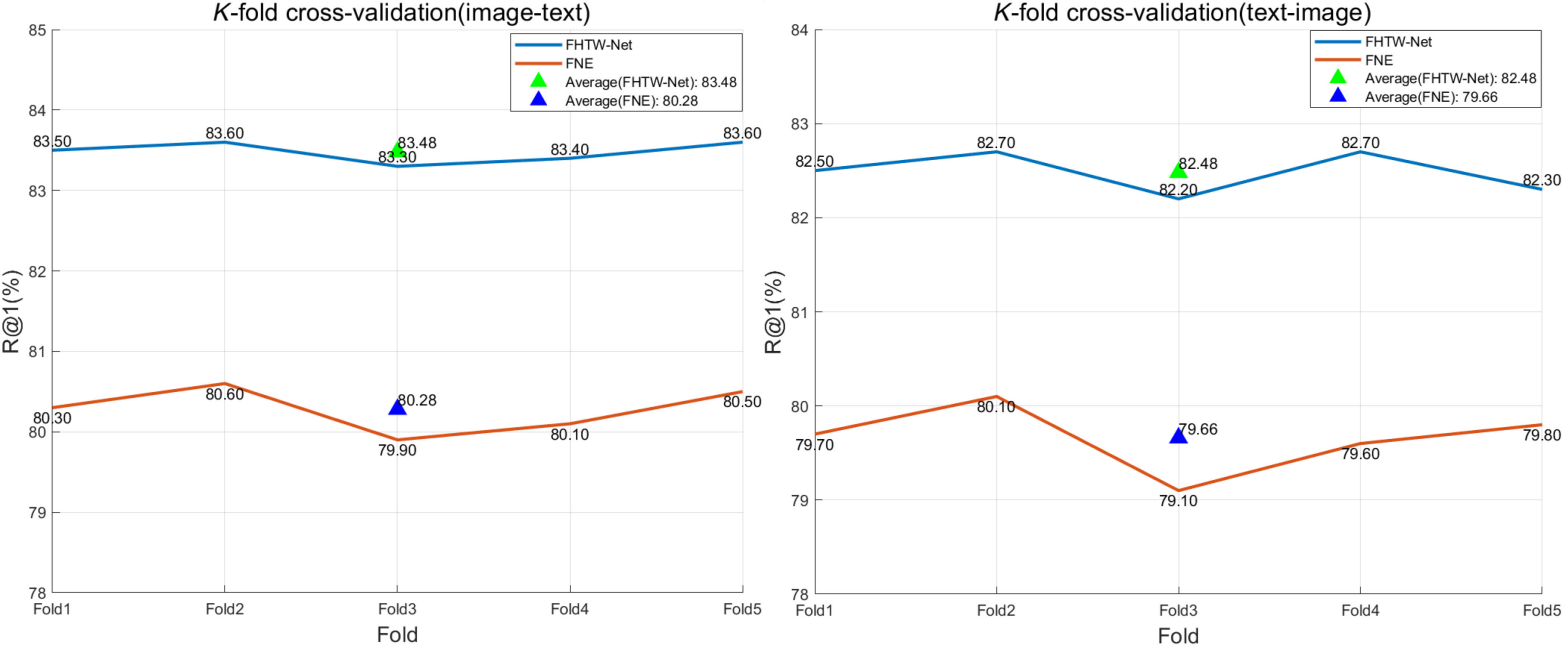
K-fold cross-validation.

Furthermore, to verify the reliability of model training, we used K-fold cross-validation [44] to improve the accuracy and reliability of the model. This is done by extracting most of the samples from specific modeling samples to set up the model, leaving a small part of the samples for prediction by the newly established model, and then determining the prediction error of this small portion of samples and recording their sum of squares. This method is repeated until all samples have been predicted once. In this paper, we used a fivefold cross-validation method, distributing 80% of the training samples and 10% of the validation samples (with the remaining 10% reserved for testing) for model validation. The accuracy of the five experiments and the average accuracy are shown in Fig. 8. From Fig. 8, under the random partitioning of training and validation sets, our FHTW-Net shows a stable improvement compared to the preimproved model FNE.

### Sensitivity analysis of hyperparameter

We conducted an experimental evaluation on the impact of different prior retrieval probabilities 𝑝 values set in Eq. 14. Given the scarcity of retrieval examples in real-world scenarios, we assigned an extremely low value to 𝑝, such as 1/10,000, and fine-tuned it using the validation set. The results are shown in Table 3. The final retrieval performance stays unaffected by varying parameter settings, even in the presence of a hundredfold disparity between 1/1,000 and 1/100,000. The use of the function showing exponential growth in generating the ultimate weights proficiently minimizes the influence of this parameter, resulting in a more consistent retrieval performance. It is seen that the model achieves best performance when 𝑝 is set to 1/10,000, and therefore, this value is consistently kept in all experiments.

**Table 3.** Sensitivity analysis results of hyperparameter 𝑝 in Eq. 14

Methods	Images to text			Text to images		
R@1	R@5	R@10	R@1	R@5	R@10
1/1,000	82.3	90.9	93.1	81.1	96.2	98.0
1/5,000	82.8	91.2	93.4	81.4	96.4	98.1
1/10,000	83.5	92.0	94.0	82.5	98.0	98.5
1/50,000	83.4	91.1	93.2	81.7	96.3	98.4
1/100,000	82.9	91.0	93.3	81.5	96.0.1	98.2

**Fig. 9.**
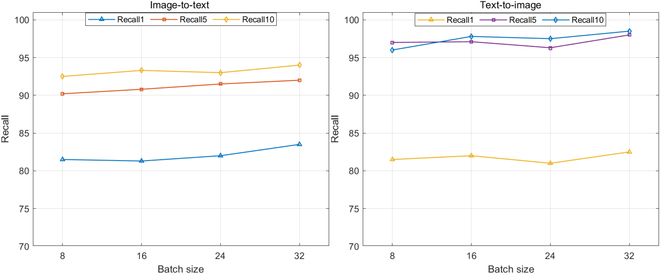
The effect of batch size on the momentum memory module.

As highlighted in earlier research [45–47], enlarging batch sizes leads to enhanced results in image–text retrieval, attributed to the abundance of negative samples. Emphasizing again, the memory unit significantly enlarges the sample reservoir, tackling the issue of constrained negative samples in smaller batches. For a more in-depth exploration of effectiveness with smaller batch sizes, we conducted experiments with a consistent memory length of 8,192, varying the batch sizes to 24, 16, and even 8. The experimental results are shown in Fig. 9. This observation reveals that models with varying batch sizes prove comparable performance, suggesting that our model is no longer affected by batch size due to the inclusion of our extensive memory module. With this improvement, our approach can run effectively with smaller batch sizes, such as eight samples per batch, even on devices with limited memory capacity.

### Performance comparison

Baseline and Existing Approaches. Assessing the effectiveness of our proposed FHTW-Net model involves a comparison with several existing technological baselines using the CRLDRD dataset, as delineated in Table 4. As outlined in Introduction, these baselines methods form:

**Table 4.** Retrieval results on the test set of rice leaf disease cross-modal image–text retrieval

Method	Images to text			Text to images		
R@1	R@5	R@10	R@1	R@5	R@10
SCAN [22]	72.6	85.2	90.9	72.7	90.1	90.5
SAEM [48]	73.2	85.8	90.6	73.4	90.8	91.2
VSRN [50]	74.6	85.4	91.2	74.1	90.3	90.7
IMRAM [49]	76.1	86.5	91.5	75.5	92.7	93.1
GSMN [51]	77.1	87.6	91.9	76.4	94.1	94.7
AOQ [45]	77.4	88.1	92.1	76.7	95.2	95.4
CAMERA [32]	78.1	87.8	92.0	77.2	94.9	95.1
DIME [24]	78.7	88.4	92.2	77.8	95.6	95.7
VSE∞ [20]	79.1	88.2	92.1	78.3	95.3	95.5
NAAF [27]	79.6	88.9	92.3	78.9	96.0	96.3
FNE [21]	80.3	89.8	92.6	79.7	96.2	96.7
Ours (FHTW-Net)	83.5	92.0	94.0	82.5	98.0	98.5

Methods for visual semantic embedding include SAEM [48], CAMERA [32], VSE∞ [20], and our baseline model FNE [21], among others.

Cross attention (CA) methods encompass SCAN [22], AOQ [45], IMRAM [49], DIME [24], NAAF [27], and so forth.

VSE methods, the first category, prove efficient retrieval in inference but are often limited by a shortage of substantial cross-modal interaction. On the other hand, CA methods, the second category, delve into detailed cross-modal interactions, resulting in improved performance. However, their widespread adoption is impeded by their significant computational cost. Nevertheless, with the increasing popularity of pretrained architectures such as BERT [33], the effectiveness disparity between these two approaches has steadily narrowed. The baseline model FNE, functioning as a visual semantic embedding method, has already reached ultramodern performance by capitalizing on the benefits of pretrained models.

Performance Comparison. The quantitative results of a comparative study between our FHTW-Net and the ultramodern models are shown in Table 4. Initially, significant improvements are seen for FHTW-Net in the R@1, R@5, and R@10 metrics over the benchmark model FNE. Specifically, for image retrieval from text, improvements were recorded as 3.2%, 2.2%, and 1.4% for R@1, R@5, and R@10, respectively, while for text retrieval from image, the improvements were 2.8%, 1.8%, and 1.8%, respectively.

Notable performance enhancements over similar technologies were exhibited by NAAF and VSE∞ within the realm of the visual semantic embedding (VSE) method and the CA method. The NAAF in CA, derived from SCAN, has made improvements in similarity computation while emphasizing on inconsistent fragment assessments. Meanwhile, the CA method prioritizes mismatched fragment assessments, considering positive and negative effects in the attention computation process, thus enhancing CA performance. These steps contribute to the outstanding performance of NAAF. Conversely, the VSE method’s VSE∞ resolves issues of gradient vanishing by determining whether to extract challenging negative samples based on the similarity between positive and negative pairs, reaching phenomenal performance from a different perspective. VSE∞, with its innovative approach, underscores the importance of extracting hard-to-handle negative samples for image–text matching. Performance is boosted by strategically extracting challenging negative samples.

Our FHTW-Net, by implementing effective innovations such as TMS, FNE-HNM strategies, and WBA, has further improved retrieval performance, surpassing existing advanced technical benchmarks on evaluation metrics. More precisely, compared to VSE∞, our FHTW-Net has improved R@1 by 4.4% and 4.2% for image retrieval from text and text retrieval from image tasks, respectively. Similarly, our FNE-HNM improved R@1 by 3.9% and 3.6% compared to NAAF.

Comprehensive experimental results confirm the performance improvement of the model by comprehensively selecting challenging and eliminating false negatives and hard-to-handle negative samples through the proposed FNE-HNM in negative sampling, thereby ensuring a comprehensive selection of negative samples. Beyond that, it resolves common false-negative issues in image–text cross retrieval tasks using our FHTW-Net, enabling the learning of precise and unique image and text feature presentations. Furthermore, the proposed TMS enhances image and text features and improves retrieval performance. Ultimately, with the use of WBA for learning rate optimization, the speed at which the way the model trains and converges is accelerated, thereby enhancing the model’s precision. The combination of the various innovative modules effectively improves the cross-modal reciprocal retrieval performance of texts and images of rice leaf disease.

## Discussion

**Fig. 10.**
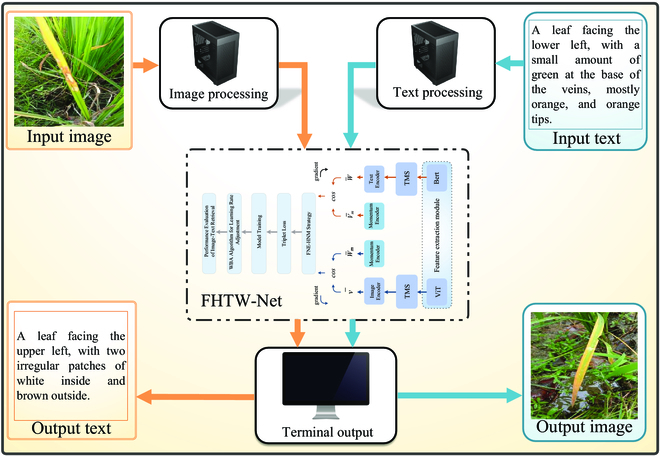
Schematic diagram of automatic rice cross-modal image–text retrieval system based on FHTW-Net.

To evaluate the effectiveness of the FHTW-Net model in cross-modal retrieval of rice leaf disease images and text, we implemented an automated system for retrieving disease-related image–text pairs. Figure 10 illustrates the architecture of this system. Initially, we gathered 400 images depicting four varieties of rice leaf diseases (namely, bacterial leaf blight, rice blast, brown spot, and tungro) by conducting field photography in Yue yang, Hunan, China. Subsequently, we annotated the images with text to ease cross-modal image–text retrieval and to confirm the accuracy of our retrieval system in retrieving either images or text. Our system has two functions: image retrieving text and text retrieving image. For the image retrieving text function, we input the image into the image processing module. The image processing module uses the FHTW-Net model to perform text retrieval on the image data. To match the model, the image size is automatically converted to 256 × 256. Finally, the input image and the retrieved text result will be displayed through the terminal, along with specific processing recommendations. After deployment, rice disease leaf images will be sent over the network to the server for text retrieval, and the retrieval text results will be promptly fed back to aid in the specific analysis and evaluation of rice disease leaf image–text retrieval. For the text retrieving image function, we input the text into the text processing module. The text processing module uses the FHTW-Net model to perform image retrieval on the text data. Finally, the input text and the retrieved image result will be displayed through the terminal, along with specific processing recommendations. After deployment, rice disease leaf text will be sent over the network to the server for image retrieval, and the retrieved image results will be promptly fed back to aid in the specific analysis and evaluation of rice disease leaf text-image retrieval.

In Table 5, we present the quantitative results for the generalization of our approach, FHTW-Net, and the baseline model, FNE, on our self-collected dataset of 400 images and annotated text pairs. Compared to the preimproved FNE, FHTW-Net shows a significant enhancement in model generalization performance in the practical application of rice leaf disease image–text retrieval. It proves a good adaptability to the real-world tasks of cross-modal image–text retrieval.

**Table 5.** Quantitative results for model generalization comparison

Method	Images to text			Text to images		
R@1	R@5	R@10	R@1	R@5	R@10
FNE [21]	79.2	89.1	92.0	78.8	95.7	96.2
Ours (FHTW-Net)	82.1	91.4	93.6	81.4	97.2	97.9

**Fig. 11.**
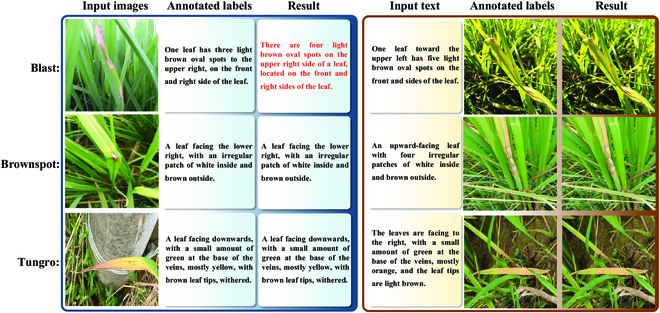
Application results of the retrieval system.

Figure 11 displays several results produced by our retrieval system. Overall, the output aligns well with our expectations. The system performs cross-modal image–text retrieval of rice leaf diseases effectively, although occasional misidentification cases still exist (an exemplar of misclassified red text). Images retrieved via text by our model match actual images quite adeptly, with rare instances of misidentification. This especially occurs when various stages of the same disease have similar text labels, suggesting that the model’s capability to retrieve the same type of disease at fine-grained stages needs enhancement. Although these occasional misidentifications do not influence the retrieval judgment of the same disease, the temporal classification issue for various stages of infection in the same leaf disease is still a considerable challenge we face.

Looking forward, to tackle the potential misidentification issues of text or images at various stages of the same disease, we are considering the establishment of a fine-grained image–text reciprocal retrieval framework for leaf disease severity. Such fine-grained considerations stem from the perspective of early prevention and control of leaf diseases. When our framework is further perfected and capable of addressing potential text or image misidentification issues at various stages of the same disease in the future, it will provide a more effective cross-modal retrieval tool for early prevention of leaf diseases. This will offer a more fine-grained control technique upgrade for prevention and control of leaf diseases.

Furthermore, for field deployment in agricultural regions for direct practical use, we consider combining Internet of Things (IoT) sensor technology with cross-modal retrieval technology in the future. Enhanced cross-modal retrieval technology can detect various stages of leaf disease infection more accurately over time. This broader approach will bolster comprehensive early warnings for diseases and enable the system to retrieve leaf diseases at various stages, hence enabling timely, targeted actions for effective disease control.

## Conclusion

We conducted research in the field of agriculture, focusing on the issue of crop diseases. Through an in-depth study of crop leaf disease detection, we proposed a cross-modal image–text retrieval approach to rapidly and accurately retrieve various aspects of information about rice leaf diseases, providing timely and precise methods for prevention and treatment: (a) By proposing TMS, we achieved more precise semantic alignment between images and text, ensuring that the model can understand and match semantic information accurately. (b) Our research has proposed the FNE-HNM strategy, addressing the issue of selecting challenging negative samples and eliminating false negatives when applied to model training using the triplet loss function. This significantly enhances the model’s robustness and generalization ability. The strategy also improves the accuracy of image–text information extraction and retrieval. Additionally, FHTW-Net delves into the semantic relationships between different modalities, further enhancing retrieval performance. (c) In the model training process, we proposed WBA for learning rate optimization, accelerating model convergence and improving retrieval accuracy. The improvement significantly enhanced the model’s retrieval performance, providing producers with the ability to retrieve timely and accurate information about rice leaf diseases. Additionally, this offers fresh insights for the application of deep learning in contemporary rice agriculture. (d) The extensive experimental results on the CRLDRD dataset show that our proposed FHTW-Net model outperforms existing ultramodern methods. In image-to-text retrieval, it achieved R@1, R@5, and R@10 accuracies of 83.5%, 92%, and 94%, while in text-to-image retrieval, it achieved accuracies of 82.5%, 98%, and 98.5%. Compared to the baseline model FNE, advanced VSE∞, and NAAF methods, our model improved R@1 accuracy by 3.2%, 4.4%, and 3.9% in image retrieval text, and 2.8%, 4.2%, and 3.6% in text retrieval image tasks. Ablation experiments proved that the introduction of modules such as TMS, HNM, and WBA significantly improved the precision of image–text retrieval, achieving more precise semantic alignment between images and text.

However, there are still issues that need to be addressed. It is essential to set up severity levels for diseases in the early warning system of disease detection. Leaf diseases have a certain development cycle, progressing with the advancement of bacterial parasitism, starting from localized infection and gradually spreading to widespread infection, disseminating throughout the entire leaf. Despite the unique features of different diseases, the same disease may show different visual characteristics at various stages of development. Conducting detailed cross-modal retrieval of leaf disease images and text features at different disease stages, and defining the severity of the disease, can provide early warnings of leaf disease outbreaks for early prevention. It also eases rapid repair and control when the disease has already developed. This approach maximally restrains leaf diseases from the initial stages, reducing production losses and unnecessary resource wastage. Additionally, in the first stages of a disease, there may be no obvious signs, and relying solely on image and text data may not be sufficient to decide whether the leaves are affected. In the future, we consider further improving and upgrading the model by integrating IoT sensor technology with cross-modal retrieval techniques. This involves further expanding multimodal data fusion to include images, text, and voice, thereby setting up a phased disease retrieval framework for the image–text–voice stages of the fine-grained disease infection process. This integration aims to enhance comprehensive early disease warning and later disease control.

## Data Availability

The partial datasets utilized and examined in this research have been posted on the website https://github.com/ZhouGuoXiong/FHTW-Net. Furthermore, for access to all bespoke datasets used in this study (comprising a total of 6,332 image–text pairs), please contact the corresponding author.
